# First report of *Anaplasma marginale* in the European bison *Bison bonasus*

**DOI:** 10.1186/s13071-025-07056-8

**Published:** 2025-11-04

**Authors:** Anna W. Myczka, Stanisław Kaczor, Elwira Plis-Kuprianowicz, Joanna Werszko, Aleksandra Kawińska, Arkadiusz Juszczyk, Anna Bajer, Zdzisław Laskowski

**Affiliations:** 1 Department of Eco-Epidemiology of Parasitic Diseases, Institute of Developmental Biology and Biomedical Sciences, Faculty of Biology, University of Warsaw, Ilji Miecznikowa 1, 02-096 Warsaw, Poland; 2 District Veterinary Inspectorate, Młynarska 45, 38-500 Sanok, Poland; 3 Białowieża National Park, Park Pałacowy 11, 17-230 Białowieża, Poland; 4 Department of General Biology and Parasitology, Medical University of Warsaw, Tytusa Chałubińskiego 5, 02-004 Warsaw, Poland; 5 Krynki Forest District, Poczopek 6D, 16-113 Szudziałowo, Poland; 6 Polish Parasitological Society, Twarda 51/55, 00-818 Warsaw, Poland

**Keywords:** *Anaplasma marginale*, *msp4*, *16S* rDNA, *Bison bonasus*

## Abstract

**Background:**

European bison, *Bison bonasus*, is a strictly protected species of large mammal, with 25% of the world’s population living in Poland. The most numerous populations of European bison live in the Białowieża Primeval Forest, northeastern Poland, and in the Bieszczady Mountains, southeastern Poland. The purpose of this study was to investigate the occurrence of *Anaplasma* spp. in *B. bonasus* from Poland.

**Methods:**

Tissue samples were collected from 45 European bisons between 2021 and 2024 in the Białowieża and Bieszczady areas. Two genetic markers, *16S* ribosomal DNA (rDNA) and *msp4*, were used for the detection, genotyping, and phylogenetic analysis of bacteria from the genus *Anaplasma*.

**Results:**

The prevalence of *Anaplasma* spp. was 40% (18/45) in the examined samples. *Anaplasma phagocytophilum* was detected in 10 samples, and eight samples were found to be positive for the presence of *Anaplasma marginale* DNA.

**Conclusions:**

This study is the first report of *A. marginale* occurrence in Poland and the first report of *A. marginale* occurrence in *B. bonasus* in Europe. Infection by the pathogenic *A. marginale* in strictly protected species such as the European bison may have an impact on the health, ecology, and conservation of this endangered species.

**Graphical abstract:**

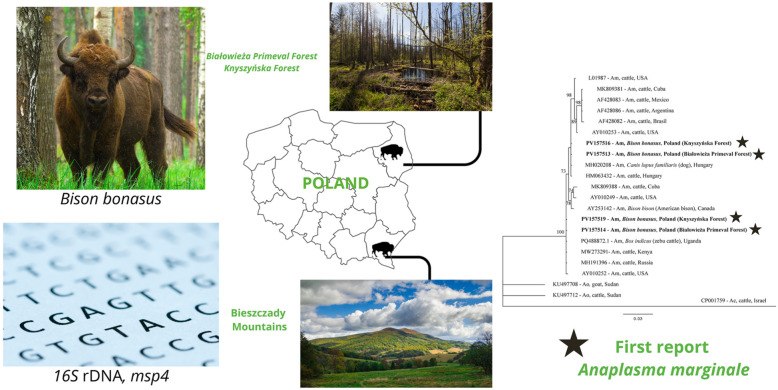

## Background

The current global population of European bison (*Bison bonasus* Linnaeus, 1758) consists of 11,180 individuals. A significant share (25%) of this population lives in Poland, mainly in two areas: the Bieszczady Mountains and the Białowieża Primeval Forest [1]. This strictly protected species was nearly extinct at the beginning of the twentieth century, but since 1919, the population has been restored with great success, including in Poland [2]. Health monitoring is currently the key aspect of species conservation and research [3, 4]. Because of a limited gene pool of European bisons, the species can be prone/susceptible to various infectious diseases [5]. The recent rapid spread of *Thelazia* spp. infection in the two largest free-living populations may be an example of such susceptibility [6].

*Anaplasma marginale* (family Anaplasmaceae, order *Rickettsiales*) is an obligate intracellular bacterium that invades erythrocytes, similar to its close relatives, *Anaplasma ovis* and *Anaplasma centrale* [7–9]. It is the most pathogenic *Anaplasma* species for cattle, but it does not infect humans [10, 11]. It is the causative agent of bovine anaplasmosis, a livestock disease with a great economic impact on dairy and cattle production [10]. The most frequently reported clinical signs are fever, anorexia, anemia, rumen atony, fetus loss, changes in urine color (dark yellow to brown), weakness, a reduction in milk production, and death in acute infection [7, 11–14].

In the literature, the endemic occurrence of *A. marginale* is strongly associated with tropical and subtropical climates [7]. However, pathogens are widely reported worldwide in North America (the USA [15], Canada [16], Mexico [17]), South America (Argentina [18], Brazil [19]), Africa (Egypt [20], Uganda [21], Zimbabwe [22]) and Asia (Malaysia [23], India [24], and Iran [25]). In Europe, probably due to climate change, the northern border of the geographical range of vectors which can transmit *A. marginale*, is moving north annually [26]. The first reports concerning European bovine anaplasmosis and the occurrence of *A. marginale* were from Mediterranean areas, such as France [27], Italy [28], Portugal [29], and Spain [30]. Cases have since been described in Central European countries such as Austria [31], Switzerland [32], and Hungary [8]. To the best of our knowledge, in Europe, there are no reports on *A. marginale* occurrence further north than the three-country (Hungary, Austria, Switzerland) line.

*Anaplasma marginale* can be transmitted between hosts by three routes: by vectors (ticks and tabanids), mechanical transfer of live infected erythrocytes (by needle pokes or surgical equipment), and through the placenta (vertical transmission) [10, 33, 34]. However, the wide distribution and great success of the spread of *A. marginale* in various habitats is due primarily to vector transmission, mainly by hard ticks from the family Ixodidae [34]. Over 20 species of ticks from three genera, *Dermacentor*, *Hyalomma*, and *Rhipicephalus*, can transmit this pathogen [9, 10, 34]. *Dermacentor reticulatus* ticks occur widely, whereas *Hyalomma* spp. occur sporadically in Poland [35, 36]; however, their role in *A. marginale* transmission in Poland is unknown. Moreover, within *Dermacentor* spp., *D. reticulatus* is not listed as a vector of *A. marginale* [34]. A few species from the family Tabanidae are also reported as vectors of *A. marginale*. This intracellular pathogen was detected in *Tabanus bovinus*, *Tabanus tergestinus*, and *Poeciloderas lindneri* in Europe and South America [37, 38]. A few of the species also occur in Poland, but *A. marginale* was not detected in them [39].

The aim of this study was to investigate the occurrence of *Anaplasma* spp. in *B. bonasus* from Poland. We determined the prevalence of infection, and genotyping and phylogenetic analyses were conducted to characterize the obtained *Anaplasma* spp. species and strains.

## Methods

### Material collection and study area

**Fig. 1:**
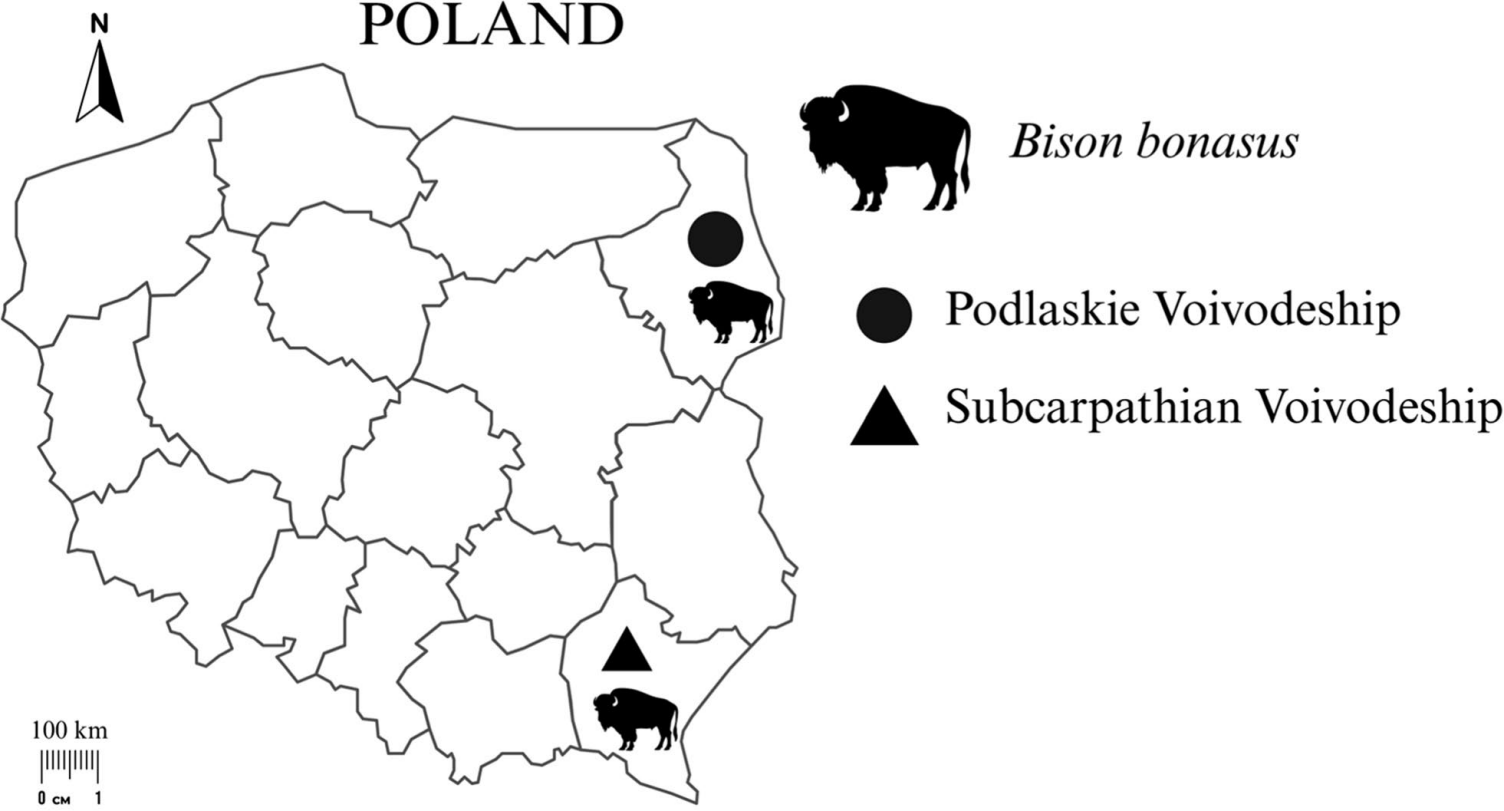
Geographical distribution of the collected *Bison bonasus* tissue samples from Podlaskie Voivodeship (NE) and Subcarpathian Voivodeship (SE)

Spleen samples were collected from 45 free-living European bison individuals. Tissue samples were collected between 2021 and 2024 at three study sites in Poland: the Białowieża Primeval Forest and the Knyszyńska Forest in Podlaskie Voivodeship in Poland (*n* = 19) and the Bieszczady Mountains in Subcarpathian Voivodeship in Poland (*n* = 26) (Fig. 1). Samples from the Podlaskie Voivodeship were taken from bisons found dead for unknown reasons and road-killed individuals (*n* = 11) or from individuals culled during selective shooting (*n* = 8) conducted by employees of the Białowieża National Park and the Knyszyńska Forest District (collection permit decisions: PN.51.10.2021 AK, DZP-WG.6401.121.2024.EB). Tissue samples from the Subcarpathian Voivodeship were collected from 26 individuals culled at the discretion of the General Directorate for Environmental Protection (collection permit decisions: DZP-WG.6401.2.2021.EB) and with the approval of the Regional Directorate for Environmental Protection in Rzeszów (collection permit decisions: WPN6401.1.194.2021.RN.2).

### *Anaplasma* spp. molecular detection

Genetic material was isolated from the spleen samples using a commercial kit for DNA isolation (DNA Mini Kit Syngen, Wrocław, Poland; and Genomic Mini AX Tissue Spin, A&A Biotechnology, Gdańsk, Poland) according to the manufacturers’ protocols.

The DNA of *Anaplasma* spp. was detected in samples by nested polymerase chain reaction (PCR) amplification and sequencing of a 400-base-pair (bp) fragment of *16S* ribosomal DNA (rDNA) according to Szewczyk et al. [40]. In samples positive for *Anaplasma* spp. DNA, partial *msp4* (major surface protein 4) gene fragments were amplified to characterize the obtained *Anaplasma* spp. An 850-bp fragment of the *msp4* gene was amplified according to de la Fuente et al. [15]. The nested PCR (for amplification of *16S* rDNA) and PCR (for amplification of *msp4*) were performed in a volume of 20 µl (2 µl of 10× PCR Dream Taq Green buffer, 0.1 µl of 5U Dream Taq, 0.2 µl of 10 mM dNTPs [Thermo Fisher Scientific, Waltham, MA, USA], 1 µl of 10 mM of each primer, and 2 µl of template DNA and filled with nuclease-free water up to 20 µl). The DNA of *Anaplasma phagocytophilum* from wild boar (GenBank accession number: MT510541) was used as a positive control [41], whereas nuclease-free water was used as a negative control.

The PCR products were visualized on a 1.5% agarose gel (EURx, Gdańsk, Poland) stained with Midori Green stain (Nippon Genetics, Düren, Germany) in the presence of a size-marked DNA marker of 100–1000 bp (A&A Biotechnology, Gdańsk, Poland). Visualization was performed by GelDoc MP Lab software (Imagine, Bio-Rad, Hercules, CA, USA). The obtained PCR products were sequenced using the Sanger method by Eurofins Genomics (Ebersberg, Germany). The sequences were aligned in CodonCode Aligner v. 11.0.1 software (CodonCode Corporation, Centerville, MA, USA). The obtained sequences were compared with the GenBank database (Basic Local Alignment Search Tool [BLAST], National Center for Biotechnology Information [NCBI], USA) and then submitted to GenBank. Phylogenetic trees of the *A. marginale msp4* partial gene (786 bp) were constructed in Bayesian inference (BI), as implemented in MrBayes version 3.2.0 software [42]. The SYM + I model was selected for the *msp4* sequences as the best-fitting nucleotide substitution model by jModelTest version 2.1.10 software [43, 44]. The analysis was run for 2,000,000 generations, with 1,000,000 generations discarded as burn-in. The phylogenetic trees were visualized in TreeView software (S&N Genealogy Supplies, UK).

## Results

Eighteen of the 45 examined samples were positive for *Anaplasma* spp., resulting in a prevalence of 40%. Of these 18 positive samples, 13 samples were from the Podlaskie Voivodeship and five from the Podkarpackie Voivodeship. Some findings regarding the detection of *Anaplasma* spp. by molecular methods have been published previously [45]. Among the 18 obtained *16S* rDNA sequences, two species were identified: *A. marginale* (*n* = 8) on the basis of 99.76–100% identity to *A. marginale* from cattle from different countries (i.e., Uganda [KU686793], Kazakhstan [PQ133434], Republic of South Africa [AF414873]), and *A. phagocytophilum* (*n* = 10) on the basis of 99.76–100% identity to *A. phagocytophilum* from Poland [ON025953], Russia [HM366579], and Estonia [MW922756]). Data concerning *A. phagocytophilum* from this study will be published elsewhere. This study is the first confirmed report of *A. marginale* in Poland and the first report of *A. marginale* in *B. bonasus* in Europe. The general prevalence of *A. marginale* was 17.8% (8/45); however, this pathogen was detected only in Podlaskie Voivodeship, and the prevalence in this area was 42.1% (8/19). *Anaplasma phagocytophilum* was detected in both study areas, and its prevalence was 22.2% (10/45).

Among the eight *A. marginale*-positive samples, two sequence variants of *16S* rDNA and *msp4* gene fragments were detected (Table 1). There was a two-nucleotide difference in the 795-bp sequence of the *msp4* gene between the two groups, namely, genotype 1 (five sequences) and genotype 2 (three sequences), resulting in 99.75% similarity between the genotypes. Five sequences of *msp4,* which formed *A. marginale* “genotype 1” (this study), showed 100% identity with the sequence of *A. marginale* from cattle, Hungary (HM063432). This Hungarian genotype was described in 2012 by Hornok et al. [8] and was associated with fatal and anaemic cases of anaplasmosis in cows in Hungary. Three *msp4* sequences that constituted “genotype 2” showed 100% identity for several *A. marginale* strains from cattle from different countries: Kenya (MW273291), Uganda (PQ488872), Russia (MH191396), and Israel (AY786993). There was only one single-nucleotide polymorphism (SNP) in the short sequences of *16S* rDNA between the two groups of sequences, resulting in 99.75% similarity between these groups.

**Table 1:** Results of the molecular analysis of *Anaplasma marginale* strains, isolated from European bison (*Bison bonasus*)

European bison sample number	GenBank accession numbers		Study site	Genotype
	*msp4*	*16S* rDNA		
Z22	PV157513^a^	PV151354	Białowieża Primeval forest	1
Z38	PV157515	PV151355	Białowieża Primeval Forest	
Z41	PV157516^a^	PV151356	Knyszyńska Forest	
Z43	PV157518	PV151357	Knyszyńska Forest	
Z45	PV223869	PV151358	Knyszyńska Forest	
Z36	PV157514^a^	PV151359	Białowieża Primeval Forest	2
Z42	PV157517	PV151360	Knyszyńska Forest	
Z44	PV157519^a^	PV151361	Knyszyńska Forest	

^a^Nucleotide sequences selected as representatives of genotype groups in sequence and phylogenetic analysis

**Fig. 2:**
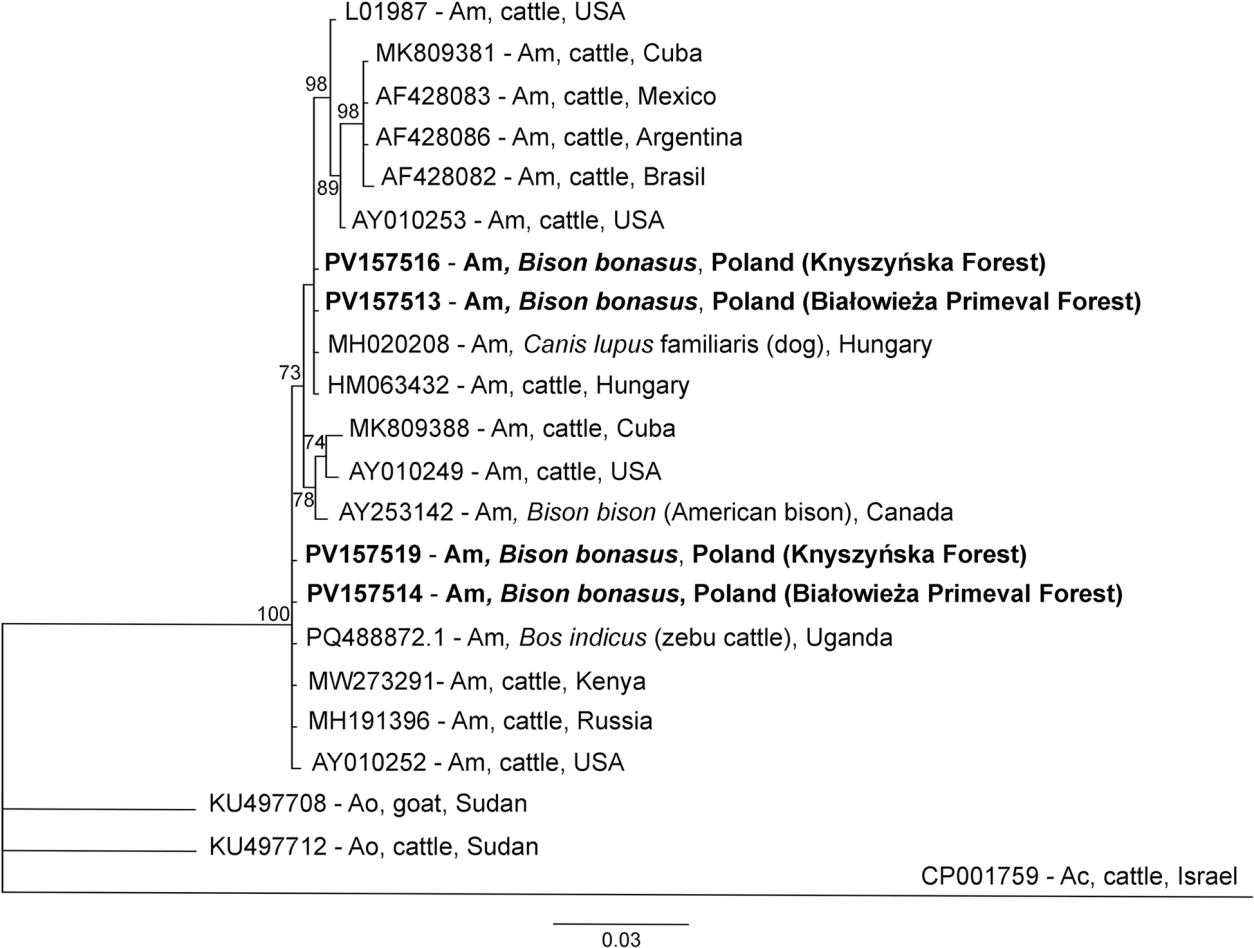
Phylogenetic tree of the *msp4* partial gene (786 bp) of *Anaplasma marginale* haplotypes constructed in Bayesian inference (BI) analysis with MrBayes version 3.2 [39]. The SYM + I model was chosen as the best-fitting nucleotide substitution model by JModelTest version 2.1.10 software [40, 41]. The analysis was run for 2,000,000 generations, with 1,000,000 generations discarded as burn-in. Nodal support is indicated as Bayesian posterior probabilities. Sequences from *A. centrale* (CP001759) and *A. ovis* (KU497708, KU497712) were used as outgroups. GenBank accession numbers, hosts, and countries of origin are shown. The sequences from this study are in bold. Am = *A. marginale*, Ao = *A. ovis*, Ac = *A. centrale*

For phylogenetic analysis, sequences of *msp4* were analysed, with 17 sequences of *A. marginale* and two root sequences of *A. ovis* and *A. centrale* from GenBank (Table 2, Fig. 2). Phylogenetic analysis of the *msp4* sequences revealed that the sequences could be divided into two clades. These two clades represented genotypes 1 and 2 and were closely related to each other. Genotypes 1 and 2 were grouped with *A. marginale* from Europe (Hungary, Russia, Moscow Region) and Africa (Uganda, Kenya), respectively (Fig. 2).

**Table 2:** Selected literature sequences (GenBank) for *msp4* partial gene phylogenetic analysis

Pathogen species	Strain name, sample origin	Accession number	Host	Reference
*Anaplasma marginale*	Oklahoma, USA	AY010252	Cattle	De la Fuente et al. [46]
	St. Maries, USA	AY010249	Cattle	De la Fuente et al. [46]
	Florida, USA	LO1987	Cattle	Wickwire et al. [47]; Allred et al. [48]
	Okeechobee, USA	AY010253	Cattle	De la Fuente et al. [47]
	Minas Gerais, Brazil	AF428082	Cattle	De la Fuente et al. [46]
	Salta, Argentina	AF428086	Cattle	De la Fuente et al. [46]
	Texcoco, Mexico	AF428083	Cattle	Garcia Ortiz et al. [49]; Garcia Ortiz et al. [50]; Rodriguez et al. [51]
	Oriente CuBov140, Cuba	MK809381	*Bos taurus* (cattle)	Díaz-Sánchez et al. [52]
	CuBov132, Cuba	MK809388	*Bos taurus* (cattle)	Díaz-Sánchez et al. [52]
	Moscow Region, Russia	MH191396	Cattle	No data
	Kilifi County, Kenya	MW273291	Cattle	No data
	Uganda	PQ488872	*Bos indicus* (zebu cattle)	Etiang et al. [53]
	Canada	AY253142	*Bison bison* (American bison)	De la Fuente et al. [54]
	Hungary	HM063432	Cattle	Hornok et al. [8]
	Hungary	MH020208	Dog	No data
*A. ovis*	Sudan	KU497708	Goat	No data
	Sudan	KU497712	Cattle	No data
*A. centrale*	Israel	CP001759	Cattle	Herndon et al. [55]

## Discussion

To the best of our knowledge, this is the first confirmed report of *A. marginale* in Poland, the first report of *A. marginale* in *B. bonasus*, and the first report of this pathogen occurring further north than Hungary, in Europe [8].

There is one report about the potential occurrence of *A. marginale* in Poland. In 2011, *Anaplasma* spp. were tested in Warmian–Masurian Voivodeship cattle by serological methods (enzyme-linked immunosorbent assay [ELISA]), and the seroprevalence of *A. marginale* was 1.7% (23/1326) [56]. However, the commercial test used in this study, the *Anaplasma* Antibody Test Kit, cELISA (VMRD, Inc., Pullman, WA, USA), which is still available on the market, enables the detection of several *Anaplasma* species in addition to *A. marginale*, as stated in the manufacturer’s description. Different species of *Anaplasma* can infect cattle, including *A. phagocytophilum*, *A. centrale*, and *A. bovis*, in addition to *A. marginale* [57]. This previous report was based solely on ELISA, and the findings were not confirmed by molecular or microscopic (smear) methods. In addition, at that time in Europe, *A. marginale* was not reported in cattle, even in Hungary, until 2009 [8].

*Bison bonasus* populations in Poland are affected by numerous parasites and bacteria, including *Ashworthius sidemi*, *Haemonchus contortus*, *Ostertagia kolchida*, *Thelazia gulosa*, *Toxoplasma gondii*, *Chlamydia* spp., and *A. phagocytophilum* [4, 6, 45, 58]. Our study has added *A. marginale* to the list of European bison pathogens. This pathogen can constitute a serious threat to the health of cattle and European bisons, which are representative of the family Bovidae. Bovine anaplasmosis is responsible for annual losses in livestock of up to 9 million euros worldwide [59]. Additionally, many fatal cases of bovine anaplasmosis are reported every year [8, 60, 61], which highlights the real threat of *A. marginale* to the *B. bonasus* population. Notably, the detection of *A. marginale* among the Polish population of the European bison is most likely an example of autochthonous infection, because the Polish population of *B. bonasus* was restored as the first one in Europe [1]. In fact, in many European countries where this species was reintroduced, Polish individuals were used to establish a population [62]. Therefore, the research we present sheds new light on aspects of *A. marginale* epidemiology, considering the transport of individual bison for reintroduction purposes, theoretically from an area where *A. marginale* was not previously present or endemic. This may indicate a potential epidemiological threat to wild and farm animals in new areas where reintroduced *B. bonasus* individuals may serve as reservoirs of *A. marginale*. In contrast to the cattle trade, which facilitates the transport of *A. marginale*-infected livestock from endemic to non-endemic regions [7], this is not the case in the Polish population of *B. bonasus.*

So far, there are no data about the prevalence of *A. marginale* in *B. bonasus*. To date, only *A. phagocytophilum*, related to *A. marginale*, has been detected in Polish and European populations of European bison [46, 63–67], with quite marked prevalence rates ranging from 18% to 67%. In the present study, the overall prevalence of *Anaplasma* spp. was 40%, 22.2%, and 17.8% for *A. phagocytophilum* and *A. marginale*, respectively, which is in line with previous reports [46, 63–67]. Interestingly, the prevalence of *A. marginale* in American bison (*Bison bison*) in US and Canadian populations is much higher, ranging from 88% to 100% [54].

An interesting aspect of our study is the occurrence of *A. marginale* in only one population of examined European bison, Podlaskie Voivodeship (Białowieża Primeval Forest and Knyszyńska Forest), and the lack of detection of this pathogen in samples from the Bieszczady Mountains. The presence or absence of *A. marginale* can be associated with the occurrence and/or availability of its vectors in the examined areas. A similar situation was described in the Argentinian population of cattle [68], where *Rhipicephalus* spp. are associated with the transmission of *A. marginale* in the environment. *Rhipicephalus microplus* occurs in northern Argentina, the area with optimal temperature and humidity for reproduction of this tick species, but it was not found in southern Argentina. A similar pattern was observed for *A. marginale* occurrence: infection was detected in the northern area but not in the southern area [68].

The vector of *A. marginale* in Poland is unknown. Three genera of ticks and flies are referred to as possible vectors: *Dermacentor*, *Hyalomma*, and *Rhipicephalus* [9, 10, 34], and *T. bovinus*, *T. tergestinus*, and *P. lindneri* [37, 38]. *Dermacentor reticulatus* (ornate dog tick), *Hyalomma* spp., and *T. bovinus* occur in Poland [35, 36, 38]. It is highly unlikely that *Hyalomma* spp. ticks are responsible for the transmission of *A. marginale* in Poland, as the first report of the occurrence of these ticks was from 2024 (unpublished data, [36]), and according to our results, this bacterium was detected in samples collected in 2021. From the family Tabanidae, only *A. phagocytophilum* was reported in examined specimens in Poland [39]. It seems more likely that *A. marginale* can be transmitted by ornate dog ticks. The eastern metapopulation of this hard tick is reported in the northwestern part of Poland (Podlaskie Voivodeship), the same region where *A. marginale* is prevalent in the examined animals. The opposite situation occurs in the southwestern region, where *D. reticulatus* is not present (Subcarpathian Voivodeship), and no positive samples for *A. marginale* were detected there [35]. In the Central European *B. bonasus* population, two species of ticks feed on bisons, *Ixodes ricinus* and *D. reticulatus* [67], but the first hard tick is not identified as an *A. marginale* vector, and in the second, *A. marginale* has not yet been detected, in either Poland or Europe. Additionally, because *A. marginale* was detected in two different areas (Białowieża National Park and Knyszyńska Forest District), there is a low probability of transmission of these bacterial pathogens other than by vector transmission. However, the occurrence of *A. marginale* in Poland has not yet been confirmed in any of the hard tick species, and the vector of transmission remains unknown.

For phylogenetic and phylogeographical analysis of *A. marginale* isolates from European bisons, fragments of the *msp4* gene were used. This genetic marker is highly conserved among *A. marginale* and is more useful and informative for this type of analysis than the *16S* rDNA gene. In addition, the *msp4* gene is frequently used in phylogenetic analysis [8, 15]. Our *msp4* sequences of genotype 1 clustered with the European strain of *A. marginale* from cattle and dogs in Hungary. The sequences of genotype 2 clustered with *A. marginale* sequences from Africa and Russia (Moscow Region). None of the genotypes obtained from this study were strongly associated with *A. marginale* strains from North and South America, which were isolated from naturally infected hosts. Our results of phylogenetic analysis seem to confirm that tropical and subtropical bacterial parasites, such as *A. marginale*, are expanding their geographical range northwards from Africa to Europe, likely in response to climate change [26].

## Conclusions

Our study provides new data about the occurrence, genetic diversity, and changes in the geographical range of *A. marginale*. Infection by the pathogenic *A. marginale* in strictly protected species such as the European bison may have an impact on the health, ecology, and conservation of this endangered species. The tick vectors and routes of *A. marginale* transmission in Poland remain unknown. The influence of climate change on the distribution of this pathogen and its vector must be investigated in the near future to understand the short- and long-term consequences for humans, wild animals, and livestock.

## Data Availability

No datasets were generated or analysed during the current study.

## References

[1] Raczyński J, Bołbot M, Księga Rodowodowa Żubrów [In Polish]. 2023. www.bpn.gov.pl/ksiega-rodowodowa-zubrow.

[2] Krasiński ZA. Żubr Puszcz Imperator [In Polish]. Wydawnictwo BPN: Białowieża, Poland. 2005. https://bpn.gov.pl/zubr-puszcz-imperator.

[3] Olech W, Klich D, Perzanowski K. Development of a new action plan for the European bison. Oryx. 2019;53:214–214. 10.1017/S0030605318001369.

[4] Didkowska A, Klich D, Hapanowicz A, et al. Pathogens with potential impact on reproduction in captive and free-ranging European bison (*Bison **bonasus*) in Poland—a serological survey. BMC Vet Res. 2021;17:345. 10.1186/s12917-021-03057-8. 34736464 10.1186/s12917-021-03057-8 PMC8567710

[5] Bielecki W, Amarowicz J, Hławiczka M, et al. Monitoring zdrowia populacji żubrów jako element ochrony gatunku [In Polish]. Eur Bison Conserv Newslett. 2014;7:43–50.

[6] Demiaszkiewicz AW, Moskwa B, Gralak A, et al. The nematodes *Thelazia gulosa* Railiet and Henry, 1910 and *Thelazia skrjabini* Erschov, 1928 as a cause of blindness in European bison *(Bison bonasus*) in Poland. Acta Parasitol. 2020;65:963–8. 10.1007/s11686-020-00243-w. 32613456 10.1007/s11686-020-00243-w PMC7679292

[7] Kocan KM, de la Fuente J, Blouin EF, et al. The natural history of *Anaplasma* marginale. Vet Parasitol. 2010;10:95–107. 10.1016/j.vetpar.2009.09.012. 10.1016/j.vetpar.2009.09.012 19811876

[8] Hornok S, Micsutka A, Fernández de Mera IG, et al. Fatal bovine anaplasmosis in a herd with new genotypes of *Anaplasma marginale*, *Anaplasma ovis* and concurrent haemoplasmosis. Res Vet Sci. 2012;92:30–5. 10.1016/j.rvsc.2010.10.011. 21094505 10.1016/j.rvsc.2010.10.011

[9] Falghoush A, Ku PS, Brayton KA. Immunization with *Anaplasma* centrale Msp2 HVRs is less effective than the live A. Centrale Vaccine against Anaplasmosis. Vaccines (Basel). 2023;29:1544. 10.3390/vaccines11101544. 10.3390/vaccines11101544 PMC10610995 37896947

[10] Aubry P, Geale DW. A review of bovine anaplasmosis. Transbound Emerg Dis. 2011;58:1–30. 10.1111/j.1865-1682.2010.01173.x. 21040509 10.1111/j.1865-1682.2010.01173.x

[11] Jurković D, Mihaljević Ž, Duvnjak S, Silaghi C, Beck R. First reports of indigenous lethal infection with *Anaplasma marginale*, *Anaplasma bovis* and *Theileria orientalis* in Croatian cattle. Ticks Tick-borne Dis. 2020;11:101469. 10.1016/j.ttbdis.2020.101469. 32723641 10.1016/j.ttbdis.2020.101469

[12] Dumler JS, Barbet AF, Bekker CP, et al. Reorganization of genera in the families Rickettsiaceae and Anaplasmataceae in the order Rickettsiales: unification of some species of *Ehrlichia* with *Anaplasma*, *Cowdria* with *Ehrlichia* and *Ehrlichia* with *Neorickettsia*, descriptions of six new species combinations and designation of *Ehrlichia equi* and “HGE agent” as subjective synonyms of *Ehrlichia phagocytophila*. Int J Syst Evol Microbiol. 2001;51:2145–65. 10.1099/00207713-51-6-2145. 11760958 10.1099/00207713-51-6-2145

[13] Atif FA. *Anaplasma marginale* and *Anaplasma phagocytophilum*: rickettsiales pathogens of veterinary and public health significance. Parasitol Res. 2015;114:3941–57. 10.1007/s00436-015-4698-2. 26346451 10.1007/s00436-015-4698-2

[14] Ben Said M, Belkahia H, Messadi L. *Anaplasma* spp. in North Africa: a review on molecular epidemiology, associated risk factors and genetic characteristics. Ticks Tick-borne Dis. 2018;9:543–55. 10.1016/j.ttbdis.2018.01.003. 29398602 10.1016/j.ttbdis.2018.01.003

[15] de la Fuente J, Van Den Bussche RA, Prado TM, Kocan KM. *Anaplasma* marginale msp1α genotypes evolved under positive selection pressure but are not markers for geographic isolates. J Clin Microbiol. 2003;41:1609–16. 10.1128/JCM.41.4.1609-1616.2003. 12682152 10.1128/JCM.41.4.1609-1616.2003 PMC153873

[16] Howden KJ, Geale DW, Paré J, Golsteyn-Thomas EJ, Gajadhar AA. An update on bovine anaplasmosis (*Anaplasma marginale*) in Canada. Can Vet J. 2010;51:837–40. 21037882 PMC2905000

[17] Rodríguez SD, García Ortiz MA, Jiménez Ocampo R, Vega y Murguía CA. Molecular epidemiology of bovine anaplasmosis with a particular focus in Mexico. Infect Genet Evol. 2009;9:1092–101. 10.1016/j.meegid.2009.09.007. 19786123 10.1016/j.meegid.2009.09.007

[18] Ruybal P, Moretta R, Perez A, et al. Genetic diversity of *Anaplasma**marginale* in Argentina. Vet Parasitol. 2009;26:176–80. 10.1016/j.vetpar.2009.02.006. 10.1016/j.vetpar.2009.02.006 19285808

[19] Silva JB, Cabezas-Cruz A, Fonseca AH, Barbosa JD, de la Fuente J. Infection of water buffalo in Rio de Janeiro Brazil with *Anaplasma**marginale* strains also reported in cattle. Vet Parasitol. 2014;15:730–4. 10.1016/j.vetpar.2014.09.009. 10.1016/j.vetpar.2014.09.009 25260335

[20] Mahmoud HYAH, Ali AO, Tanaka T. Molecular detection and characterization of *Anaplasma**marginale* infecting cattle, buffalo, and camel populations in southern Egypt. Front Vet Sci. 2023;11:1169323. 10.3389/fvets.2023.1169323. 10.3389/fvets.2023.1169323 PMC10213943 37252392

[21] Byaruhanga C, Collins NE, Knobel DL, Khumalo ZTH, Chaisi ME, Oosthuizen MC. Molecular detection and phylogenetic analysis of *Anaplasma marginale* and *Anaplasma centrale* amongst transhumant cattle in north-eastern Uganda. Ticks Tick Borne Dis. 2018;9:580–8. 10.1016/j.ttbdis.2018.01.012. 29422446 10.1016/j.ttbdis.2018.01.012

[22] Norval RA, Fivaz BH, Lawrence JA, Brown AF. Epidemiology of tick-borne diseases of cattle in Zimbabwe. II. Anaplasmosis. Trop Anim Health Prod. 1984;16:63–70. 10.1007/BF02239846. 6485099 10.1007/BF02239846

[23] Ola-Fadunsin SD, Gimba FI, Abdullah DA, Sharma RSK, Abdullah FJF, Sani RA. Epidemiology and risk factors associated with *Anaplasma marginale* infection of cattle in Peninsular Malaysia. Parasitol Int. 2018;67:659–65. 10.1016/j.parint.2018.06.013. 29960083 10.1016/j.parint.2018.06.013

[24] Ntesang K, Singla LD, Kaur P, Arora JS, Kashyap N. Molecular epidemiology, phylogenetic analysis and risk assessment of *Anaplasma marginale* from naturally infected bovines of Punjab (India). Acta Trop. 2022;232:106499. 10.1016/j.actatropica.2022.106499. 35523271 10.1016/j.actatropica.2022.106499

[25] Yousefi A, Rahbari S, Shayan P, Sadeghi-Dehkordi Z, Bahonar A. Molecular detection of *Anaplasma**marginale* and *Anaplasma**ovis* in sheep and goat in west highland pasture of Iran. Asian Pac J Trop Biomed. 2017;7:455–9. 10.1016/j.apjtb.2017.01.017.

[26] Baylis M. Potential impact of climate change on emerging vector-borne and other infections in the UK. Environ Health. 2017;16:112. 10.1186/s12940-017-0326-1. 29219091 10.1186/s12940-017-0326-1 PMC5773876

[27] Poncet A, Chossonery A, Brugère-Picoux J. L’anaplasmose bovine. Bull Soc Vét Prat de France T. 1987;71:381–400.

[28] de la Fuente J, Torina A, Caracappa S, Tumino G, et al. Serologic and molecular characterization of *Anaplasma* species infection in farm animals and ticks from Sicily. Vet Parasitol. 2005;5:357–62. 10.1016/j.vetpar.2005.05.063. 10.1016/j.vetpar.2005.05.063 16043300

[29] Caeiro V. General review of tick species present in Portugal. Parassitologia. 1999;1:11–5. 11071535

[30] de La Fuente J, Vicente J, Höfle U, Ruiz-Fons F, et al. *Anaplasma* infection in free-ranging Iberian red deer in the region of Castilla-La Mancha, Spain. Vet Microbiol. 2004;3:163–73. 10.1016/j.vetmic.2004.02.007. 10.1016/j.vetmic.2004.02.007 15145495

[31] Baumgartner W, Schlerka G, Fumicz M, Stöger J, et al. Seroprevalence survey for *Anaplasma**marginale*-infection of Austrian cattle. Zentralbl Veterinarmed B. 1992;39:97–104. 10.1111/j.1439-0450.1992.tb01143.x. 1621479 10.1111/j.1439-0450.1992.tb01143.x

[32] Hofmann-Lehmann R, Meli ML, Dreher UM, Gönczi E, et al. Concurrent infections with vector-borne pathogens associated with fatal hemolytic anemia in a cattle herd in Switzerland. J Clin Microbiol. 2004;42:3775–80. 10.1128/JCM.42.8.3775-3780.2004. 15297529 10.1128/JCM.42.8.3775-3780.2004 PMC497630

[33] Baldacchino F, Desquesnes M, Mihok S, Foil LD, Duvallet G, Jittapalapong S. Tabanids: neglected subjects of research, but important vectors of disease agents! Infect Genet Evol. 2014;28:596–615. 10.1016/j.meegid.2014.03.029. 24727644 10.1016/j.meegid.2014.03.029

[34] Rar V, Tkachev S, Tikunova N. Genetic diversity of *Anaplasma* bacteria: twenty years later. Infect Genet Evol. 2021;91:104833. 10.1016/j.meegid.2021.104833. 33794351 10.1016/j.meegid.2021.104833

[35] Karbowiak G. Changes in the occurrence range of hosts cause the expansion of the ornate dog tick *Dermacentor**reticulatus* (Fabricius, 1794) in Poland. Biol. 2022;77:1513–22. 10.1007/s11756-021-00945-0.

[36] Romanek W, Bajer A. Narodowe Kleszczobranie—Nauka Obywatelska. [in Polish]. (2025). the promising start of a citizen science project. Parasites Vectors 18:383. 10.1186/s13071-025-07022-4www.narodowekleszczobranie.pl. 10.1186/s13071-025-07022-4 PMC12462190 40993823

[37] Rodrigues GD, Lucas M, Ortiz HG, Dos Santos GL, et al. Molecular of *Anaplasma**marginale* Theiler (Rickettsiales: Anaplasmataceae) in horseflies (Diptera: Tabanidae) in Uruguay. Sci Rep. 2022;28:22460. 10.1038/s41598-022-27067-0. 10.1038/s41598-022-27067-0 PMC9797482 36577829

[38] Hornok S, Földvári G, Elek V, et al. Molecular identification of *Anaplasma**marginale* and rickettsial endosymbionts in blood-sucking flies (Diptera: Tabanidae, Muscidae) and hard ticks (Acari: Ixodidae). Vet Parasitol. 2008;4:354–9. 10.1016/j.vetpar.2008.03.019. 10.1016/j.vetpar.2008.03.019 18495345

[39] Werszko J, Szewczyk T, Steiner-Bogdaszewska Ż, et al. Molecular detection of *Anaplasma**phagocytophilum* in blood-sucking flies (Diptera: Tabanidae) in Poland. J Med Entomol. 2019;16:822–7. 10.1093/jme/tjy217. 10.1093/jme/tjy217 30615168

[40] Szewczyk T, Werszko J, Myczka AW, et al. Molecular detection of *Anaplasma**phagocytophilum* in wild carnivores in north-eastern Poland. Parasites Vectors. 2019;12:465. 10.1186/s13071-019-3734-y. 31590678 10.1186/s13071-019-3734-y PMC6781336

[41] Myczka AW, Szewczyk T, Laskowski Z. The occurrence of zonotic *Anaplasma phagocytophilum* strains, in the spleen and liver of wild boars from north-west and central parts of Poland. Acta Parasitol. 2021;66:1082–5. 10.1007/s11686-021-00368-6. 33770340 10.1007/s11686-021-00368-6 PMC8390417

[42] Ronquist F, Huelsenbeck JP. MrBayes 3: Bayesian phylogenetic inference under mixed models. Bioinformatics. 2003;19:1572–4. 10.1093/bioinformatics/btg180. 12912839 10.1093/bioinformatics/btg180

[43] Guindon S, Gascuel OA. Simple, fast, and accurate algorithm to estimate large phylogenies by maximum likelihood. Syst Biol. 2003;52:696–704. 10.1080/10635150390235520. 14530136 10.1080/10635150390235520

[44] Darriba D, Taboada GL, Doallo R, Posada D. Jmodeltest 2: more models, new heuristics and parallel computing. Nat Methods. 2012;9:772. 10.1038/nmeth.2109. 22847109 10.1038/nmeth.2109 PMC4594756

[45] Myczka AW, Kaczor S, Filip-Hutsch K, Czopowicz M, Plis-Kuprianowicz E, Laskowski Z. Prevalence and genotyping of *Anaplasma phagocytophilum* strains from wild animals, European bison (*Bison bonasus*) and Eurasian moose (*Alces alces*) in Poland. Animals. 2022;12:1222. 10.3390/ani12091222. 35565648 10.3390/ani12091222 PMC9105415

[46] de la Fuente J, Van Den Bussche RA, Garcia-Garcia JC, Rodríguez SD. Phylogeography of New World isolates of *Anaplasma**marginale* based on major surface protein sequences. Vet Microbiol. 2002;88:275–85. 10.1016/s0378-1135(02)00122-0. 12151201 10.1016/s0378-1135(02)00122-0

[47] Wickwire KB, Kocan KM, Barron SJ, Ewing SA, Smith RD, Hair JA. Infectivity of three *Anaplasma**marginale* isolates for *Dermacentor**andersoni*. Am J Vet Res. 1987;48:96–9. 3826850

[48] Allred DR, McGuire TC, Palmer GH, Leib SR, et al. Molecular basis for surface antigen size polymorphisms and conservation of a neutralization-sensitive epitope in *Anaplasma marginale*. Proc Natl Acad Sci U S A. 1990;87:3220–4. 10.1073/pnas.87.8.3220. 1691504 10.1073/pnas.87.8.3220 PMC53867

[49] Ortiz MA, Ojeda LE, Salgado GH, et al. Caracterización de la virulencia de un aislado mexicano de *Anaplasma**marginale* Tec. Pecu Mex. 1998;36:202.

[50] Ortiz MA, Torres RA, Salgado GH, Alarcón JG, Rodríguez SD. *Anaplasma**marginale*: diferentes grados de virulencia en dos aislados mexicanos. Vet Méx. 2000;31:157–60.

[51] Rodríguez SD, Garcîa Ortiz MA, Hernández Salgado G, Santos Cerda NA, et al. *Anaplasma**marginale* inactivated vaccine: dose titration against a homologous challenge. Comp Immunol Microbiol Infect Dis. 2000;23:239–52. 10.1016/s0147-9571(99)00076-4. 11038126 10.1016/s0147-9571(99)00076-4

[52] Díaz-Sánchez AA, Meli ML, ObregónÁlvarez D, et al. Development and application of a multiplex TaqMan^®^ real-time qPCR assay for the simultaneous detection of *Anaplasma**marginale* and *Theileria**annulata* and molecular characterization of *Anaplasma**marginale* from cattle in Western Cuba. Ticks Tick Borne Dis. 2020;11:101356. 10.1016/j.ttbdis.2019.101356. 31870635 10.1016/j.ttbdis.2019.101356

[53] Etiang P, Kamusiime M, Wamala H, et al. Prevalence, genetic diversity and co-infection patterns of selected tick-borne haemoparasites infecting cattle in Karamoja region, northeastern Uganda. Res Square. 2024. 10.21203/rs.3.rs-5396831/v1.

[54] de La Fuente J, Golsteyn Thomas EJ, van den Bussche RA, Hamilton RG, et al. Characterization of *Anaplasma marginale* isolated from North American bison. Appl Environ Microbiol. 2003;69:5001–5. 10.1128/AEM.69.8.5001-5005.2003. 12902301 10.1128/AEM.69.8.5001-5005.2003 PMC169112

[55] Herndon DR, Palmer GH, Shkap V, Knowles DP Jr, Brayton KA. Complete genome sequence of *Anaplasma marginale* subsp*. centrale*. J Bacteriol. 2010;192:379–80. 10.1128/JB.01330-09. 19854912 10.1128/JB.01330-09 PMC2798241

[56] Szweda W, Siemionek J, Lipińska E, Błaszczak U, Michałowska M. Ocena prewalencji zakażeń *Anaplasma marginale* u bydła w regionie Warmii i Mazur przy użyciu cELISA MSP-5. Med Wet. 2011;67:838–42 (**in Polish**).

[57] Altay K, Erol U, Sahin OF, Aytmirzakizi A. First molecular detection of *Anaplasma* species in cattle from Kyrgyzstan; molecular identification of human pathogenic novel genotype *Anaplasma capra* and *Anaplasma phagocytophilum* related strain. Ticks Tick Borne Dis. 2022;13:101861. 10.1016/j.ttbdis.2021.101861. 34773849 10.1016/j.ttbdis.2021.101861

[58] Gałązka M, Filip-Hutsch K, Klich D, Olech W, Anusz K, Pyziel AM. The variety of abomasal nematode communities of captive and free-roaming populations of European bison, *Bison bonasus* (L.): a morphometric and molecular approach. Parasitology. 2024;151:1175–84. 10.1017/S003118202400088X. 39523646 10.1017/S003118202400088X PMC11894010

[59] de la Fuente J, Gortázar C, Contreras M, et al. Anti-tick vaccine in Uganda-from bench to field. Vaccine. 2025;25:126695. 10.1016/j.vaccine.2024.126695. 10.1016/j.vaccine.2024.126695 39765365

[60] Jaswal H, Balm MS, Singlam LD, Gupta K, Brar AP. Pathological observations on clinical *Anaplasma marginale* infection in cattle. J Parasit Dis. 2015;39:495–8. 10.1007/s12639-013-0384-4. 26345059 10.1007/s12639-013-0384-4 PMC4554589

[61] Moraga Fernández A, Ortiz JA, Jabbar A, Ghafar A, et al. Fatal cases of bovine anaplasmosis in a herd infected with different *Anaplasma marginale* genotypes in southern Spain. Ticks Tick Borne Dis. 2022;13:101864. 10.1016/j.ttbdis.2021.101864. 34775293 10.1016/j.ttbdis.2021.101864

[62] Pucek Z. European Bison (*Bison **Bonasus*): Current State of the Species and Strategy for Its Conservation. Strasbourg France: Council of Europe; 2004.

[63] Matsumoto K, Grzeszczuk A, Brouqui P, Raoult D. *Rickettsia raoultii* and *Anaplasma phagocytophilum* in *Dermacentor reticulatus* ticks collected from Bialowieza Primeval Forest European bison (*Bison bonasus bonasus*), Poland. Clin Microbiol Infect. 2009;15:286–7. 10.1111/j.1469-0691.2008.02238.x. 19548992 10.1111/j.1469-0691.2008.02238.x

[64] Scharf W, Schauer S, Freyburger F, et al. Distinct host species correlate with *Anaplasma phagocytophilum ankA* gene clusters. J Clin Microbiol. 2011;49:790–6. 10.1128/JCM.02051-10. 21177886 10.1128/JCM.02051-10 PMC3067700

[65] Dzięgiel B, Adaszek Ł, Krzysiak M, Skrzypczak M, et al. The occurrence of *Anaplasma phagocytophilum* in wild bison from the Bialowieza Primeval Forest in Eastern Poland. Berl Munch Tierarztl Wochenschr. 2015;128:310–4. 26281444

[66] Karbowiak G, Víchová B, Werszko J, et al. The infection of reintroduced ruminants—*Bison bonasus* and *Alces alces*—with *Anaplasma phagocytophilum* in northern Poland. Acta Parasitol. 2015;60:645–8. 10.1515/ap-2015-0091. 26408585 10.1515/ap-2015-0091

[67] Lipatova I, Černevičienė D, Griciuvienė L, Ražanskė I, et al. *Anaplasma phagocytophilum* in European bison (*Bison bonasus*) and their ticks from Lithuania and Poland. Ticks Tick Borne Dis. 2023;14:102246. 10.1016/j.ttbdis.2023.102246. 37639831 10.1016/j.ttbdis.2023.102246

[68] Pérez AE, Guillemi EC, Sarmiento NF, Cantón GJ, Farber MD. *Rhipicephalus**microplus* and its impact on *Anaplasma**marginale* multistrain infections in contrasting epidemiological contexts. Pathogens. 2025;7:160. 10.3390/pathogens14020160. 10.3390/pathogens14020160 PMC11858485 40005535

